# The brain tumor not-for-profit and charity experience of COVID-19: reacting and adjusting to an unprecedented global pandemic in the 21st century

**DOI:** 10.1093/noajnl/vdaa166

**Published:** 2020-12-07

**Authors:** Christina Amidei, Jean Arzbaecher, Mary Ellen Maher, Christine Mungoshi, Rosemary Cashman, Stuart Farrimond, Carol Kruchko, Chris Tse, Maureen Daniels, Sharon Lamb, Anita Granero, Mary Lovely, Jenifer Baker, Sally Payne, Kathy Oliver

**Affiliations:** 1 Department of Neurological Surgery, Feinberg School of Medicine, Northwestern Medicine, Chicago, Illinois, USA; 2 Brain Tumor Center, Department of Neurology and Rehabilitation, University of Illinois, Chicago, Illinois, USA; 3 Lou and Jean Malnati Brain Tumor Institute, Robert H Lurie Comprehensive Cancer Center, Feinberg School of Medicine, Northwestern Medicine, Chicago, Illinois, USA; 4 Zimbabwe Brain Tumour Association, Harare, Zimbabwe; 5 BC Cancer Agency, Vancouver, British Columbia, Canada; 6 International Brain Tumour Alliance (IBTA), Tadworth, UK; 7 Central Brain Tumor Registry of the United States (CBTRUS), Hinsdale, Illinois, USA; 8 The Gerry & Nancy Pencer Brain Tumour Centre, Princess Margaret Cancer Centre, University Health Network, Toronto, Canada; 9 Oscar’s Angels, Toulouse, France and Rome, Italy; 10 National Brain Tumor Society, Newton, Massachusetts, USA

**Keywords:** brain tumor organization, COVID-19, support services

## Abstract

**Background:**

The Coronavirus Disease 2019 (COVID-19) pandemic has affected individuals as well as disease-specific brain tumor organizations. These organizations around the world exist to address unmet needs for patients and caregivers they serve. The direct impact of the pandemic on these organizations constitutes significant collateral damage. In order to better understand the effects of the COVID-19 pandemic on brain tumor organizations, the International Brain Tumour Alliance (IBTA) carried out an international survey to identify organizational changes induced by the virus and approaches adopted to address challenges.

**Methods:**

A 37-question online survey consisting of categorical and qualitative questions was developed and circulated to 130 brain tumor organizations across the world. Seventy-seven organizations from 22 countries completed the survey (59% return rate). Descriptive statistics and content analysis were used to present the results.

**Results:**

Responses fell into the following 3 categories: (1) organizational characteristics, (2) impact of COVID-19 on services, and (3) COVID-19 impact on financial and human resources within organizations. Although organizational characteristics varied, common concerns reported were activity disruption which impacted organizations’ abilities to offer usual services and challenges to sustaining funding. Both financial and human resources were stressed, but integral adaptations were made by organizations to preserve resources during the pandemic.

**Conclusions:**

Although brain tumor organizations have been impacted by the COVID-19 pandemic, organizations quickly adjusted to this unprecedented global healthcare crisis. Nimble reactions and flexibility have been vital to organization sustainability. Innovative approaches are required to ensure organizations remain viable so that needs of brain tumor community at large are met.

**Key Points**

- Brain tumor organizations experienced funding challenges due to the COVID-19 pandemic.
- Brain tumor support services shifted online in response to COVID-19 pandemic challenges.

**Importance of the Study**

This is the first international survey completed with the purpose of identifying the impact of the coronavirus pandemic on brain tumor charities and not-for-profit organizations that support the brain tumor community. Surveys were sent to 130 organizations with 77 responding from 22 countries. Common stressors and concerns were identified that represent the real-world experience of these organizations. Highlighted are adaptations to the crisis that supported organizational viability. While financial and human resources were stressed, integral adaptations were made by organizations to preserve resources during the pandemic. This article provides parallel information to an article that surveyed the patient and caregiver experience.

Severe acute respiratory syndrome coronavirus 2 (SARS-CoV-2), more commonly known as coronavirus, has raged across the globe for at least the last 8 months (and possibly longer) with a ferocity that has stunned the world. Coronavirus disease 2019 (COVID-19) is the disease caused by infection with SARS-CoV-2, the classical symptoms of which include a self-limiting cough, fever, fatigue, and loss of smell or taste. Many of those infected with SARS-CoV-2 apparently experience few or no ill effects, while others experience life-threatening acute respiratory distress syndrome. On March 11, 2020, the coronavirus was declared a pandemic by the World Health Organization (WHO).^1^ As of September 10, 2020, there have been more than 27 million cases of COVID-19 confirmed worldwide and nearly 900,000 deaths.^2^ Much has been written about the effects that COVID-19 has had on global healthcare systems, economies, and populations.^3–5^ Between January and May 2020, it was been estimated that greater than 23,000 scientific COVID-19 papers were published, with the number doubling every 20 days.^6^ However, the indirect and long-lasting impact of the pandemic is yet to be realized.

Brain tumor not-for-profits and charities (“brain tumor patient organizations”) around the world exist in all shapes and sizes and address unmet needs for the patients and caregivers they serve. Some provide education, support, and information. Others raise crucial funds for brain tumor research or raise awareness of the challenges of the disease. Other such organizations are more policy-orientated and work at the governmental level to improve the situation and outcomes for brain tumor patients and their caregivers. Some brain tumor patient organizations undertake a number of these activities and some undertake all of them.

Brain tumor not-for-profits and charities have always provided a crucial lifeline to people with or affected by a brain tumor. During the COVID-19 pandemic, patients and caregivers have had to rely even more heavily on these organizations for support and information.^7^ However, the pandemic has also meant that these organizations have had to quickly adapt to relocating staff and volunteers to their homes; rapidly upskilling digital competencies and developing new methods of delivering services; cancelling vital fund-raising activities and suffering substantial drops in income, leading to concerns for their future sustainability.

The direct impact that the pandemic has had on these organizations and the people they serve constitutes significant collateral damage. In order to better understand the effect that the COVID-19 pandemic has had on brain tumor patient organizations, the International Brain Tumour Alliance (IBTA) conducted an international survey to determine how and if these organizations were able to cope with the changes brought about by the virus and how they are dealing with the numerous challenges COVID-19 has created for their day-to-day operations.

## Methods

An anonymous online survey was developed by the IBTA and circulated to 130 brain tumor patient organizations across the world. The 37-question survey ran from May 6, 2020 to June 1, 2020 and it comprised a combination of categorical and qualitative, open-ended questions (Table 1). In total, 77 organizations from 22 countries completed the survey, a return rate of 59%. Numbers of responses for each question varied as organizations could choose whether or not to respond to each question.

**Table 1.** Survey Questions

Organization Characteristics	Number of Responses
In what country is the organization registered as a legal entity?	66
What is your organization’s constitutional status?	67
In what country is your organization physically based (this might be the same country in which your organization is register or it might be a different country)?	67
In what parts of the world do you support patients and provide information to them?	66
How long has your organization been running?	67
Approximately how many patients/caregivers does your organization provide support and information to at this time?	67
How many paid employed staff members do you have?	67
How many volunteers do you have?	67
Do you fundraise from the general public in order to sustain your organization?	66
Are you supported with funding from any of the following sources whether or not you fundraise from the general public?	63
What are your main areas of activity?	67
What is your organization’s approximate average annual income—the equivalent of:	65
Does your organization do any of the following?	43
Are there any other comments you would like to make?	20
Impact	
As an organization, what are your biggest fears about the COVID-19 pandemic? Please rank the following fears with 1 being your worst and 10 being your least fear.	53
Have you made any contingency plans to cover for staff and volunteer sickness or self-isolation during this time?	53
Have you had or will you have to cancel or postpone any fundraising, conference, campaigning or meeting events this year as a result of the COVID-19 pandemic?	52
If you have been or will be forced to cancel or postpone any fundraising, conference, campaigning or meeting events this year, have you been able to run these instead as virtual/webinar events?	53
On a scale of 0–100 how would you rate the level of disruption that cancelling or postponing an event has caused your charity or not-for-profit?	53
On a scale of 0–100 how anxious are you personally about the COVID-19 pandemic specifically with regards to the sustainability of your charity or not-for-profit?	51
Have you had to permanently let paid staff go as a result of COVID-19?	53
What is the biggest challenge you have with keeping your supporters engaged with your organization at this COVID-10 pandemic time?	44
How much has your personal workload increased since the COVID-19 pandemic began?	52
What are the major issues related to COVID-19 being reported to you by your brain tumor patient communities?	51
COVID-19 has created a lot of uncertainty and turbulence for everyone including of course, all of those in the brain tumor community, no matter what their role is. In your opinion, what, if any, positive outcomes at your charity or not-for-profit might result from the challenges of the COVID-19 pandemic?	41
Resources	
Would you like/benefit from further support/training for providing online/virtual alternatives of live events/meetings?	52
What online meeting platforms does your organization use for its virtual activities during COVD-19?	52
Has your national government set up any programs or funding streams to provide financial support to charities and not-for-profits struggling with the challenges of COVID-19?	53
If your government offers financial compensation to charities and not-for-profits would you apply for a grant to help you through this COVID-19 crisis?	51
How have you adapted your charity/not-for-profit work during this COVID-19 pandemic?	49
How well informed do you feel about COVID-19 generally?	53
How well informed do you feel about COVID-19 and brain tumors specifically?	52
Who or what is your main source of medical information regarding COVID-19 in terms of your work at your charity or not-for-profit?	51
Are there any other sources of information that you use to educate yourself (and potentially your work colleagues and patients/families) about COVID-19?	31
What kind of additional information about COVID-19 would you like to see at this time?	28
In terms of being of service to the community of brain tumor patients and caregivers which you serve, what is your top priority right now in the midst of the COVID-19 crisis?	47
Once this current pandemic is over and you are able to take stock if there are any gaps in your services as a result of COVID-19, would you be willing to work with another organization and join forces to fill this gap?	52

Responses were then grouped into the following 3 categories: (1) organizational characteristics, (2) the impact of COVID-19 on services provided by brain tumor patient organizations, and (3) the impact of COVID-19 on financial and human resources for brain tumor patient organizations. Descriptive statistics were used to analyze categorical questions where appropriate. Content Thematic Analysis^8^ was used to extract themes from the qualitative data obtained from the open-ended questions, with text responses reviewed by at least 3 reviewers to assure themes were accurately captured.

## Results

### Organizational Characteristics

#### Geographic

Survey responses (*N* = 66) were received from 3 geographical regions (Table 2): (1) Americas (North/Central/South America, *N* = 26, 39%); (2) Europe (*N* = 23, 35%); and (3) Africa/Asia/Oceania (*N* = 17, 26%). Of the organizations which responded to the survey, 55% described themselves as not-for-profit, 36% were described as charities, 2% were identified as otherwise uncategorized limited companies, while 7% were other types of groups, such as a Facebook group and a volunteer committee in a brain tumor center. Of the responding brain tumor patient organizations, the majority (84%) have been in existence for more than 5 years while only 2 organizations have been functioning for less than 1 year. Whether the length of time the organization has been in existence relates to their overall resiliency during the COVID-19 crisis is yet to be seen.

**Table 2.** Number of Responding Organizations by Country (*N* = 66)

Country	Number of Responding Organizations
Australia	6
Belgium	1
Cameroon	1
Canada	3
Croatia	1
Cyprus	1
Denmark	1
France	1
India	1
Ireland	1
Italy	3
Japan	2
Lithuania	1
New Zealand	3
Norway	1
Poland	1
Singapore	1
South Africa	1
Sweden	3
United Kingdom	9
United States	23
Zimbabwe	1
Total	66

While 77 organizations responded, only 66 provided country of origin; no data are available for countries not responding to the survey.

#### Patient support

Several questions in the survey related to organizational factors pertaining to patient support. In response to these questions, brain tumor patient organizations demonstrated a broad reach. Most organizations surveyed (73%) provide support to the brain tumor community in their own country, while 26% provide support internationally. Only 1% of brain tumor patient organizations surveyed support patients regionally.

The annual number of patients and caregivers supported by surveyed brain tumor organizations varies (*N* = 67). Twenty-two percent of organizations (*N* = 15) surveyed support up to 100 people annually; 25% of organizations (*N* = 17) support between 101 and 300 people annually; 16% (*N* = 11) of organizations support between 301 and 1000 people. Nineteen percent (*N* = 13) of those organizations surveyed support between 1001 and 5000 people annually, 3% (*N* = 2) support 5001–10,000 people, and 10% (*N* = 6) support more than 10,000 people each year. Three organizations (5%) were unaware of the population size they served.

#### Activities

Those brain tumor patient organizations which responded to the survey are involved in multiple activities. Their primary activities are: providing information and support (*N* = 56, 84%); fundraising for patient support (*N* = 38, 57%); offering website and digital communications (*N* = 36, 54%); fundraising for brain tumor research (*N* = 35, 52%); and campaigning and raising awareness about brain tumors (*N* = 35, 52%). Additionally, some organizations run brain tumor support group meetings (*N* = 30, 45%); organize brain tumor conferences for patients and caregivers (*N* = 19, 28%); support policy work (*N* = 16, 24%); volunteer in hospitals or hospice settings (*N* = 8, 12%); promote pediatric initiatives (*N* = 8, 12%); and provide online webinars (*N* = 8, 10%). Additional activities include offering scholarships; connecting brain tumor survivors; providing family camps; collaborating with other organizations; and collecting and analyzing population-based information.

In addition to the activities that directly support brain tumor patients and caregivers, organizations also identified various other roles. These included providing patient or organizational representation on boards and task forces (63%); partnering with other disease groups (51%); participating in initiatives outside the cancer field (44%); co-authoring scientific papers (26%); advising on clinical trial design (23%); advising the pharmaceutical industry (16%); and advising regulatory agencies (12%). Twenty-one percent of organizations identified other activities in which they were involved, such as helping to connect researchers with each other; collaborating with physicians; and participating in medical conferences.

#### Staff and volunteers

Staffing levels in the brain tumor organizations which responded to the IBTA survey varied. Most organizations identified a combination of paid and volunteer staff members. Almost half of the organizations (46%) had no paid staff and 40% had between 1 and 4 paid staff members. The remaining organizations (14%) had between 5 and 50 paid staff members. Organizations with very few paid staff members relied on volunteers to support their missions. Survey responses indicated that volunteer capacity in brain tumor patient organizations ranged from 1 to 4 people (15%); 5 to 10 people (22%) 11 to 20 people (24%), 21 to 35 people (10%), 36 to 50 people (4%); and greater than 50 people (22%). One organization surveyed did not use any volunteers.

Contingencies for staff or volunteer absences due to illness with the COVID-19 virus or as a result of the lockdown were planned by less than half of the organizations. Workloads were shifted among staff members, or were either delayed or simply went undone, particularly when individuals were ill or worried about contracting COVID-19. Others could not perform their jobs due to extra family obligations, such as providing childcare or home schooling, that were a consequence of COVID lockdowns.

#### Finances

The remaining organizational-focused questions in the survey related to the financial situation of the organizations. With regard to average annual income (all amounts in US Dollars), 29% of the organizations reported less than $25,000 annual income, with 23% reporting between $101,000 and $300,000 in annual income, and 14% between $51,000 and $100,000. Ten organizations (15%) reported their average annual income is above $1,000,000.

Organizations were asked about their sources of financial support. A large percentage (86%) reported relying on fundraising from the general public. Apart from funding from the general public, the survey also sought to explore other sources of funding brain tumor patient organizations utilized. Of the 77 organizations which responded to the survey, 63 answered this question. Apart from donations from the general public, the most common other funding source was from other charitable trusts and foundations (60% of organizations relied on these sources) followed by conducting special events (59%). Other funding sources included tribute memorial funds (22%); membership fees (19%); pharmaceutical company support in the form of grants and donations (18%); legacies (16%); lottery funding (6%); and donations from professional institutions (11%). Only 2% of respondents received statutory funding (ie, from central government, local authorities, etc.).

There were general concerns from most organizations about maintaining financial viability and resource mobilization during the COVID-19 period and even beyond. These concerns were described as “…not knowing whether we will be able to hold our main fundraising event in October”; ...“how do we keep funds coming in through tough times when people have lost jobs etc.”; “…not holding our May event…where we raise 50% of our funds....” There was also a growing realization that the pandemic situation may be prolonged, and that the post-COVID-19 era may be different in many ways from the pre-COVID period.^9^

### Impact of the Coronavirus Pandemic on Services Provided by Brain Tumor Patient Organizations

In the IBTA survey, brain tumor patient organizations expressed concerns about the long-term impact that COVID-19 may have on their current services (Table 3). Understanding and quantifying the effect that COVID-19 has on their ability to continue these vital activities is crucial. The 2 greatest concerns expressed were the following: (1) experiencing a substantial drop in funding and (2) not being able to provide optimal services to brain tumor patients and their families.

**Table 3.** Organizations’ Greatest Fears About the COVID-19 Pandemic and How It Affects Them

Greatest Fear	*N* (%)
Experiencing a substantial drop in funding	20 (40)
Not being able to provide optimal services to brain tumor patients and their families	15 (30)
Having to modify our 2020 work program	7 (14)
Having to permanently lay off some staff members	2 (4)
Not being able to meet commitments regarding our charity’s or not-for-profit’s funding of current research projects	1 (2)
A reduction of staff availability due to self-isolation of being diagnosed with COVID-19	1 (2)
Losing volunteers and other supporters	1 (2)
Having to close our operations temporarily during the COVID-19 pandemic	1 (2)
Having to close our organization permanently as a result of the COVID-19 pandemic	1 (2)
Paying bills to suppliers	0 (0)

These concerns arose primarily because of the disruption of the organizations’ activities by COVID-19 and the consequences of abrupt cessation of activities because of sudden virus lockdowns imposed in various countries. Fundraising events, conferences, and meetings were cancelled by 94% of the brain tumor patient organizations which responded to the survey, with 39% cancelling 5 or more events because of the pandemic. Event cancellation meant that organizations faced the threat of not being able to raise the funds necessary to sustain their activities or provide the usual patient and caregiver information and support that is a substantial part of their organizational mission.

Brain tumor patient organizations cited several reasons for challenges from COVID-19 in maintaining strategic funding levels. Beyond fundraising events being cancelled, organizations reported that monies that would normally be given to brain tumor patient organizations were instead being diverted to organizations that supported activities directly related to COVID-19. In addition, communities and individuals who often provided financial support for brain tumor not-for-profits and charities found themselves in financial crises as well, and funding in general became less available. For those brain tumor patient organizations in settings with limited resources already, funding decline was even more devastating, with more than half of those organizations surveyed concerned about their sustainability. However, having to close their doors permanently as a result of COVID-19 was the greatest concern for only one organization, which speaks well of the resiliency and determination of the responding organizations overall.

Maintaining engagement of their stakeholders during the COVID-19 era was reported as a struggle for many organizations. The in-person activities often used to engage stakeholders, such as retreats, workshops, or conferences, were suddenly cancelled, resulting in limited opportunities for patients, caregivers, and supporters to interact face to face. The lack of personal interaction during the COVID-19 quarantine also strained engagement and decreased organizations’ productivity, particularly as organizational communication patterns changed while individuals worked from home. Recruiting new members or supporters was also constrained by lockdowns. Information provided to constituents in the brain tumor community also changed, with 63% of organizations stating that they were asked to provide specific information to their brain tumor communities about COVID-19.

Organizations worried about having to modify current programs due to the pandemic and quickly realized the need to create and maintain a greater online presence by adapting their programming. For some organizations, with this realization also came acknowledgement of their lack of technical literacy. As organizations’ events were cancelled, only 25 (47%) organizations were able to provide those activities as virtual events. Fear of technology, lack of technological support needed to create webinars and online support programs, and unreliable internet were several challenges cited by organizations in the survey. One organization commented about security and privacy concerns as a downside of virtual meetings: “when having an ‘open’ meeting, one cannot be quite sure who is really attending.” Online security and privacy remained a concern, even with password protected entry into virtual meetings. The shift to using technology to deliver what had originally been in-person programs left organizations feeling apprehensive about losing the personal touch in their work and the dynamism and vitality that comes from face-to-face meetings. One respondent described the change as “moving from high-touch to high-tech.”

According to the organizations responding to the survey, 71% reported that individuals in their brain tumor constituencies feared contracting COVID-19 while attending doctor visits or being hospitalized. Sixty-nine percent of brain tumor patient organizations said that in the communities which they serve, patients and caregivers were concerned about treatments being delayed, cancelled, modified, or substituted because of the pandemic. Sixty-nine percent of organizations surveyed also reported that caregivers feared they would contract COVID-19 and were concerned they would be unable to provide care. Not surprisingly, organizations felt that initially they were largely unprepared to address these concerns, but quickly learned to adapt.

Organizations reported a shift in concerns in the brain tumor community that were uniquely induced or exacerbated by the pandemic (Table 4). Increased anxiety was the most significant issue patients or caregivers reported to organizations (92% of organizations responding to the survey reported this issue), followed by concerns about isolation while locked down/quarantined (71%), which limited care and support provided at home. However, a few organizations received feedback from the brain tumor community that described social isolation as having a positive effect: “During the lockdown it was so quiet, no rush. That is good for brain tumor patients.” Another organization commented that they noted brain tumor patients were already used to social isolation and being at home a lot, and they did not personally feel too heavily impacted by lockdown restrictions. One organization commented that in discussions with brain tumor patients during lockdown, many people felt that it was nice to “have all the family under one roof for a change.”

**Table 4.** Major COVID-19 Issues Reported to Brain Tumor Not-for-Profit and Charitable Organizations Around the World by Brain Tumor Patients and Caregivers

Issues Reported	*N* (%)
Generally, greater anxiety	47 (92)
Pressures of self-isolation (ie, missing the help and companionship of other family members who are not allowed to visit because of self-isolation rules or transport restrictions)	36 (71)
Fear of going to in-person hospital or clinic appointments and being infected with COVID-19	36 (71)
Treatment delays, modifications, substitutions and/or cancellations	35 (69)
For the patient, fear of being diagnosed with COVID-19	35 (69)
For the caregiver, fear of being diagnosed with COVID-19 and becoming unable to look after the patient	32(63)
Additional childcare pressures (such as home-teaching)	25 (49)
Loss of employment for the caregiver	25 (49)
Additional burden on caregivers due to COVID-19	25 (49)
Concerns of patients that hospital staff where they are being treated do not have sufficient personal protective equipment	19 (37)
Concerns about meeting financial commitments such as mortgage repayments, other loans, insurance policies, etc.	19 (37)
Transportation issues (ie, getting to hospital or clinic where public transport has been severely curtailed or stopped due to the threat of COVID-19)	19 (37)
Challenges with participating in clinical trials/participation in clinical trials delayed or cancelled	18 (35)
End of life issues	16 (31)
Substantial difficulty contacting clinical teams regarding medical concerns	16 (31)
Increased or newly emerging mental health challenges	14 (27)
Loss of employment for the patient	13 (25)
Being redirected to a different treatment centre	12 (24)
Food shortages	12 (24)
Problems with the supply of medicines/shortages of medicines	11 (22)
Significant difficulty in organizing food deliveries or click and collect services at the local supermarket	8 (16)
Issues with medical insurance and reimbursement	6 (12)
Other concerns	5 (10)
Being asked to complete a DNR (do not resuscitate) order although at this time a patient is doing well	3 (6)
Concerns about fake medicines offered online to treat COVID-19	1 (2)

Brain tumor patient organizations also noted positive outcomes from the impact of the pandemic. As organizations began to adjust to the “new normal,” qualitative responses in the survey highlighted that many organizations were hopeful about their futures. Some found time during lockdown to resolve issues that they did not have time to address previously, and tackled projects they had planned to start in the future. Others were forced to re-evaluate and subsequently regenerate their funding mechanisms, and some found ways to save money by spending differently. New ways of working to accomplish goals and achieve the organizational mission were realized. As an example, one organization increased the frequency of their online support group meetings, and they were able to reach individuals they had not been able to reach prior to the pandemic. Others found success in maximizing the use of technology to remove barriers and enhance access to their programs. The pandemic also presented opportunities for some organizations to develop new relationships they had not previously imagined, finding strength through collaboration. Many organizations expressed appreciation for the IBTA survey, indicating it created a sense of unity of purpose among organizations, as well as giving them the opportunity to reflect on the impact of the pandemic on their work. One comment was: “Having the results of this survey and the patient/caregiver survey will help us advocate for support from our governments and from our funders. It will also open up new partnerships which will make the brain tumor community more effective.” The patient/caregiver survey to which this respondent refers is a survey which the IBTA also carried out, as part of its work with the Society for Neuro-Oncology (SNO) COVID-19 Task Force and was an international survey on how COVID-19 had affected brain tumor patients and caregivers around the world.”

### How COVID-19 Has Affected Financial and Human Resources in Brain Tumor Patient Organizations

There were several survey questions focusing on financial and human resources. Organizations were asked how they were managing financially and whether they were equipped with appropriate technology to deal with lockdowns and subsequent restrictions on face-to-face meetings.

The survey asked if organizations were equipped to provide virtual meetings. Of 52 responders to this question, half said that they would benefit from additional support to run technology or virtual services properly; the other half indicated that they had sufficient staff to be able to set up and run these technologies. The most-utilized platform for virtual meetings was Zoom at over 80% and then Microsoft Teams at 23%. Several organizations mentioned that the patients they serve do not have the technology that would support these applications, such as internet access.

The survey asked whether organizations received government support during COVID-19, with the understanding that government support would vary across the globe. Of 53 organizations responding to this question, 49% indicated that they had received COVID-19 funding from their government. Others indicated that as they were not frontline employers, such as hospitals, other health care facilities, or first responders, government funding did not apply to them. Some survey respondents indicated that they had applied to a furlough program, a job keepers program, or payroll protection program to help their employees, with government funding providing loans to small businesses allowing employee payroll support for up to 8 weeks. Those organizations that did receive funding were able to continue to provide services for up to 3 months, but thereafter they would have to cut staff or services.

Successfully sustaining brain tumor patient organizations went beyond staff and payroll protection. The IBTA survey revealed that the loss of fund-raising activities limited organizations’ resources to provide patient support and fund research. The survey enquired if organizations would be applying for government grants to make up for the shortfall caused by the loss of fundraising activities. Over 60% indicated that they would apply for financial compensation from their government if eligible. Almost 40% indicated they would not apply for such grants. Some responders indicated that they required these types of grant monies for basics such as rent and technology support, while others said they would use the money to provide specific services, such as meals or transportation, to patients or their families during treatment or hospitalizations. Concerns were also expressed about applying for loans that would place organizations in debt in order to cover current, unanticipated expenses due to COVID-19.

Brain tumor patient organizations were asked to identify how they communicated internally with colleagues during the pandemic. Many indicated changes in formats for delivering information to fellow workers, with 63% of those organizations responding that digital platforms and telephone conferencing were used to conduct new and previously planned face-to-face internal management and team meetings as well as for networking sessions with external stakeholders/funders and medical professionals.

Additional internal changes that organizations had to make included a shift to staff members working from home, sometimes with more limited resources than in their office workspace. Specific limitations reported include lack of computer video access, not having a printer readily available, or poor internet services.

Acquiring donations for continuing brain tumor research became a significant challenge for some organizations. Some brain tumor not-for-profits and charities had to shift research funding toward better understanding of the impact of COVID-19 on brain tumor patients. Funding also shifted away from research toward patient support services. For example, organizations reported funding shifts toward providing more online services, including access to support groups, surveys and general information. Organizations essentially learned to maximize the use of technology to remain relevant during a time when face-to-face contact was not possible.

Almost all survey respondents (82%) indicated that they are now well informed about COVID-19 in general. No organization responded that it did not feel informed at all. The survey queried the responders about their most trusted source of information. Fifty-one percent ranked government agencies and 41% ranked the doctor as their most trusted sources of COVID information. When asked specifically about COVID-19 information that they would like to see at this time, 80% of organizations indicated that they did not need additional information. Others responded that they needed direct information related to the impact of COVID-19 on brain tumor patients specifically, while other organizations said they wanted information regarding the impact of COVID-19 on treatment delays or changes.

One brain tumor patient organization reported that it was feeling very isolated and wondered how other organizations were coping. Some organizations expressed concern that their patients did not have adequate access to technology or were not able to use it to their advantage. This was particularly evident as brain tumor patients and caregivers were increasingly forced to use technology to communicate with their medical professionals or to reach out to their support organizations. The long-term effects of COVID-19 on public policy and the health system in general pose additional concerns, especially around access to care for people with brain tumors. The ability of caregivers to cope and advocate for their family member when not able to attend hospital or clinic visits with their loved ones was another issue.

### Priorities

The survey asked an open-ended question about each organization’s top priorities for patients and caregivers during the COVID-19 pandemic with the response themes falling into the following 3 categories: (1) providing information, (2) emotional support, and (3) financial support. Some organizations reported that their main priority was focusing on helping brain tumor patients and their families who were reaching out for information especially on how COVID-19 might affect them, and for support so as to feel connected with others. One responder described these priorities: “To be there to support patients and families and provide HOPE. And be available to them for emotional support and financial support to help them cope.” Another responder identified the need to provide “support [so] that no one feels left out and alone.” Other organizations reported that they were providing emotional support for patients experiencing hospitalizations during the COVID outbreak. In terms of prioritizing financial support, several organizations were focused on actively seeking donations so that their research funding could continue as well as to provide direct financial support for patients.

## Discussion

The international brain tumor community is supported by patient organizations across the world that offer myriad services. These services promote quality of life, provide information and support, fund vital research, raise awareness, and advocate for policy change and implementation.

Fewer organizations responded from the Africa/Asia/Oceania region (*N* = 17, 26%) than the Americas (North/Central/South America, *N* = 26, 40%) and Europe (*N* = 23, 35%). This finding most likely reflects the somewhat patchy distribution of brain tumor support organizations across the world and relative lack of resources in some geographical areas. Similar geographic results were also found in a huge COVID-19 survey (*The Voice of Charities Facing COVID-19 Worldwide)*,^2^ which involved 544 organizations from 93 countries) on how all different types of these organizations across the globe (covering many sectors of society, not just healthcare) were affected by and reacting to the pandemic.^2^ Most of the charities and not-for-profits which completed “*The Voice*…” survey were from North America and Europe.

Brain tumor patient organizations vary widely in their size, funding sources, and services offered. Most organizations provide services on a national level, creating a patchwork of support that patients and families can utilize. With the global COVID-19 pandemic creating economic crises as well as health crises, it is not surprising that organizations are very concerned about financial viability during these turbulent times and being able to sustain their services in the longer term. Similar to “*The Voice…”*^2^ survey, the greatest immediate impact on brain tumor not-for-profits and charities was a decrease in financial contributions from the general public and other funding sources. Financial viability may be of particular concern for organizations that primarily rely on paid staff, while organizations that use volunteer staff may fare better but find it challenging to locate volunteers during the pandemic. In the IBTA survey, this abrupt, and sometimes severe, reduction in funding has led organizations to develop novel approaches to seeking financial support, but there remains an underlying current of deep concern about sustainability and having to offer fewer services as a result. Innovative collaborations and partnerships across organizations may be the key to addressing concerns about sustainability in the current COVID-19 and post-COVID-19 eras.

The COVID-19 pandemic has created uncertainty about many things, with one of the greatest concerns being about how and when face-to-face activities—such as brain tumor conferences, awareness-raising walks, and other social events—may be resumed based on infection risk and rates. During the height of the pandemic, most brain tumor patient organizations cancelled activities and had to divert financial and human resources elsewhere. Organizations also found it challenging to continue to engage their stakeholders in ways with which they had been familiar. Developing or increasing their online presence was the approach developed consistently across organizations to address these challenges. However, the degree of success in transitioning to that online presence varied among organizations, and in part appeared to depend on previous expertise and access to technology to support that presence. Those organizations which did not have a ready online presence when COVID-19 struck learned to quickly “adapt and adopt,” embracing the need to use technology to provide activities formerly presented in person. Morris reported on the benefits of using technology to establish and maintain relationships and found adaptation over time to be critical to its success.^9^ Organizations indicated their constituents had limited or no access to even the basic technologies to enhance communication. Those organizations need support in leveraging alternative methods to reach those who need support services but who cannot access the emerging technology used to deliver these services in the COVID-19 era.

The long-term impact of COVID-19 on brain tumor research funding is yet unknown. Not-for-profit organizations and charities contribute a significant component of funding for neuro-oncological research. Not only are research funds being diverted elsewhere, but charitable organizations that provide research funding may expect loss of donor income, as well as industry and governmental grants as a consequence of the pandemic.^13^ The impact of the COVID-19 pandemic on moving cancer care forward may not be understood for a long time.

As expected, resource availability and utilization varied across organizations. The COVID-19 pandemic has brought many issues to light for each organization, including financial challenges and/or technology applications. The IBTA survey revealed that many organizations are currently struggling yet are hopeful about their futures. Developing innovative collaborations that maximize resource sharing could be one way of supporting sustainability of these organizations. The IBTA survey also revealed that almost 80% of organizations indicated that they would be willing to work with another organization to meet needs in gap areas in the COVID-19 and post-COVID-19 eras. This survey did not provide data about how this arrangement could be specifically accomplished, and further investigation into this type of initiative is warranted.

With challenges often come opportunities, and organizations supporting the brain tumor community have found positive ways forward as they navigate the uncertain times which COVID-19 has created. Innovation is the key to successful transitions. Organizations found new ways to advocate, fundraise, and maintain online support and presence for the brain tumor community. Many organizations plan to preserve these new approaches in the future as they have found them to be practical and successful solutions to some of the challenges caused by the pandemic. Assessing acceptability and effectiveness and addressing challenges as they arise have been reported as key measures to promoting preservation of these new approaches in adapting to a new reality, and should be part of any planned approach moving forward.^10^

Results of this survey of not-for-profit and charitable organizations that support the brain tumor community are strikingly similar to those found in another survey of organizations that support the community with various types of cancer.^13^ Common findings included funding issues, concerns about meeting patient needs and isolation from constituents.

Many questions remain unanswered, and the true impact of COVID-19 on brain tumor patients has yet to be analyzed, for example, long-term outcomes, quality of life, and the cost (both financial and human) of social isolation. This highlights the need to further urgently but carefully evaluate the true collateral damage from COVID-19 on the international brain tumor community.

### Limitations of the Survey and Recommendations for the Future

The response rate of this survey was reasonable at 59%. This survey was only conducted in English, which may have posed a language barrier to those for whom English was not their native language. What is not known is why 41% of organizations approached did not respond to the survey. It could be that organizations did not respond because of increased workload due to the pandemic, survey fatigue, or other reasons. Input from those organizations may have provided different information resulting in a response bias that does not reflect the true impact on these organizations or could have further supported information presented here.

The survey was a mix of structured and open-ended questions in an attempt to capture as much of the true impact of COVID-19 on brain tumor not-for-profits and charities as possible. The survey authors recognize, however, that the COVID-19 pandemic and its effects on the brain tumor patient and caregiver community are highly complex and therefore, further investigation of some of the results in this survey is warranted. Open-ended questions were liberally answered and did provide additional insight. Questions were structured so that respondents could skip a question if they chose which led to variable response numbers for each question and some missing data.

At the time this IBTA COVID-19 survey was conducted, lockdown was a common and critical factor in the conduct of brain tumor patient organization activities and fund raising. However, lockdown was not a cure for COVID-19. With the loosening of lockdowns in a number of countries, and with the continuing threat of virus spikes and second waves, everyone must still be on their guard and remain very careful. This need for continuing vigilance has given rise to a range of even more complex questions that brain tumor not-for-profits and charities are now facing. For example, how, when, and to what extent are these organizations able to resume holding events for fundraising or face-to-face meetings? Until there is a genuine method of avoiding COVID-19 or treating it successfully, what is an acceptable degree of risk for patient organizations working with a highly vulnerable brain tumor population?

This is one of the reasons that the IBTA is recommending a follow-up survey to further investigate and understand the continuing COVID-19 situation as it pertains to brain tumor patient organizations. Such a survey could enquire about the status of these organizations and provide information about how they are continuing to cope and adjust to the many challenges which COVID-19 will undoubtedly continue to create as the pandemic evolves. In a follow-up survey, and with the benefit of hindsight, it would also be interesting to know how these organizations would have navigated the first months of the pandemic differently.

Importantly, information gleaned from a follow-up survey could be used to help form the framework for a future mitigation strategy should the world ever experience a devastating and far-reaching event like COVID-19 again. It is crucial that we in the international brain tumor community take heed now of the lessons learned about how to react to a sudden and traumatic world event that upends current practices and threatens society as a whole. Such a mitigation strategy should be planned in concert with the international brain tumor patient organization community, also focusing on issues raised in the IBTA’s first COVID-19 survey about the effects of the virus on brain tumor patients and caregivers.^6^ Such a mitigation strategy could also include an analysis of efforts that focus on promoting sustainability and leveraging technology in times of crisis. The aim of such a strategy would be to minimize disruption to brain tumor patients and caregivers; help rapidly adjust the management of these patients in terms of treatment and support; reduce fear and create confidence in the brain tumor community; and ensure that no patient becomes an unintentional victim of a pandemic when they are already battling a brain tumor.

## Conclusion

It is clear from the results of the IBTA’s survey on “*The Brain Tumour Not-for-Profit and Charity Experience of COVID-19*” that quickly reacting and adjusting to an unprecedented global healthcare crisis in the 21st century has been crucial for the viability and sustainability of brain tumor patient organizations. Once the dust settles on this turbulent and challenging time for everyone, it will be vital to ensure—should there be another worldwide crisis—that we are prepared. The challenges of brain tumors and the devastation they cause to patients and their families do not stop, even during a pandemic. Therefore, support services and information provision; raising funds for vital research into the causes of and treatments for brain tumors; raising awareness and focusing on policy work in our communities cannot stop either. We must also ensure—should there be future pandemics or world crises—that brain tumor patient organizations have a plan to not only survive these crises but continue to thrive through them and beyond, for the sake of the communities, they serve around the world.

COVID-19 has brought widespread illness, death, and suffering. However, it is important, too, that we should all additionally acknowledge that COVID-19 has also resulted in the creation of some highly innovative partnerships; previously unheard-of rapid timescales for the development of new treatment approaches; many examples of the rediscovery of community spirit; and a greater appreciation of quality time spent with family and friends. Just as the negative aspects of COVID-19 should inform our actions moving forward, so too should these positive aspects and learnings.

As for the future, the IBTA proposes a collaborative “Crisis Mitigation Strategy for Brain Tumour Patient Organisations” which would involve all interested brain tumor not-for-profits and charities who wish to create a safety net for their organizational activities in times of world crises. The IBTA invites brain tumor patient organizations, large and small, to create a forum in which current COVID-19 challenges, and future global threats, can be discussed and in which plans and guidelines can be formulated for avoiding some of the devastation that the pandemic has wreaked on our communities. We would also, as mentioned above, carefully consider the positive advances that have resulted from living through a pandemic. Brain tumor not-for-profits and charities are invited to contact kathy@theibta.org at the IBTA if they are interested in being a part of this initiative.

## Notes

**Authorship Statement.** Design of the survey and question selection: K.O., S.F., and IBTA Senior Advisors; Dissemination of survey: K.O., S.F., and IBTA Senior Advisors; Collection of all survey responses: K.O. and S.F. Analysis of all survey responses: C.A., J.A., and M.E.M. Manuscript preparation: C.A., J.A., M.E.M., K.O., C.M., and R.C. All authors read, edited, and approved the manuscript.

## Notes

Jean Arzbaecher, Mary Ellen Maher, Christine Mungoshi, Rosemary Cashman, Stuart Farrimond, Carol Kruchko, Chris Tse, Maureen Daniels, Sharon Lamb, Anita Granero, Mary Lovely, Jenifer Baker and Sally Payne are all Senior Advisors for the International Brain Tumour Alliance (IBTA).
